# Integrative Analysis Identified MCT4 as an Independent Prognostic Factor for Bladder Cancer

**DOI:** 10.3389/fonc.2021.704857

**Published:** 2021-08-26

**Authors:** Yang Zhao, Bin Zhao, Wei-Hua Yan, Yan Xia, Zhi-Hui Wang, Guo-Yang Zheng, Wen-Da Wang, Yu-Shi Zhang

**Affiliations:** ^1^Department of Urology, Peking Union Medical College Hospital, Chinese Academy of Medical Sciences and Peking Union Medical College, Beijing, China; ^2^Department of Breast Surgery, The First Affiliated Hospital, Zhejiang University School of Medicine, Hangzhou, China; ^3^Department of Pathology, Affiliated Hospital of Qingdao University, Qingdao, China; ^4^Department of Pathology, Qilu Hospital, Shandong University, Qingdao, China; ^5^Clinical College, Qingdao University, Qingdao, China

**Keywords:** bladder cancer, monocarboxylate transporter 4, solute carrier family 16 member 3, single-cell RNA sequencing, immunohistochemistry

## Abstract

**Background:**

Bladder cancer is the 10th most common cancer and most common urothelial malignancy worldwide. Prognostic biomarkers for bladder cancer patients are required for individualized treatment. Monocarboxylate transporter 4 (MCT4), encoded by *SLC16A3* gene, is a potential biomarker for bladder cancer because of its crucial role in the lactate efflux in the aerobic glycolysis process. We aimed to study the association between MCT4 expression and the overall survival (OS) of bladder cancer patients.

**Methods:**

The published single-cell RNA sequencing data of 49,869 bladder cancer cells and 15,827 normal bladder mucosa cells and The Cancer Genome Atlas (TCGA) bladder cancer cohort data were used to explore the mRNA expression of SLC16A3 in bladder cancer. Eighty-nine consecutive bladder cancer patients who had undergone radical cystectomy were enrolled as a validation cohort. The expression of MCT4 proteins in bladder cancer specimens was detected using immunohistochemistry staining. The Kaplan–Meier survival analysis and Cox regression were performed to analyze the association between MCT4 protein expression and OS in bladder cancer patients.

**Results:**

SLC16A3 mRNA was upregulated in bladder cancer cells. The upregulated genes in SLC16A3-positive epithelial cells were enriched in the glycolysis process pathway and monocarboxylic acid metabolic process pathway. Patients with high SLC16A3 mRNA expression showed significantly poor OS (p = 0.016). High MCT4 protein expression was also found to be an independent predictor for poor OS in bladder cancer patients (HR: 2.462; 95% CI: 1.202~5.042, p = 0.014). A nomogram was built based on the results of the multivariate Cox analysis.

**Conclusion:**

Bladder cancer with high SLC16A3 mRNA expression has a poor OS. High MCT4 protein expression is an independent prognostic factor for bladder cancer patients who had undergone radical cystectomy.

## Introduction

Bladder cancer is the 10th most common cancer and most common urothelial malignancy worldwide, with approximately 573,000 new cases and 213,000 deaths per year (1). Radical cystectomy is the first-line treatment for resectable muscle invasive bladder cancer (MIBC) and is also used as radical treatment for high-risk non-MIBC (NMIBC) (2). However, 26.9%–37.5% of patients who had undergone radical cystectomy showed tumor recurrence or metastasis after surgery (3–5). Prognostic biomarkers for bladder cancer patients are needed for more individualized surveillance and intervention after surgery.

In contrast to normal cells, which rely on oxidative phosphorylation to generate the energy needed for cellular processes, cancer cells rely on glycolysis for the energy needed for survival and proliferation (6). This phenomenon is termed aerobic glycolysis or the Warburg effect, which is common among human malignancies (6, 7). Aerobic glycolysis could help tumor cells generate ATPs quickly and acquire substrates for anabolism, which is critical for tumor cell proliferation (8). Additionally, the lactate generated from aerobic glycolysis can be transported into the tumor microenvironment by membrane monocarboxylate transporters, which can re-program the infiltrated immune cells and attenuate their anti-tumor immune response (9). The intensity of tumor aerobic glycolysis may be closely related to a high malignant potential and poor survival (10).

Monocarboxylate transporter 4 (MCT4) is a member of solute carrier family 16 encoded by *SLC16A3* gene (11). It is widely expressed, particularly in tissues that rely on glycolysis for energy metabolism, such as tumor cells, immune cells, and astrocytes (12). In tumor cells, the efflux of lactate mediated by MCT4 is essential to maintain cytoplasmic pH (12, 13). The expression of MCT4 is correlated with the clinical outcome of several urologic cancers. Choi et al. (14) found that elevated MCT4 expression is associated with the incidence of castration-resistant prostate cancer and an earlier time to relapse. Fisel et al. (15) reported that the MCT4 mRNA is upregulated in renal cancer and that high MCT4 protein expression is correlated with poor overall survival (OS) in renal cancer. Previous studies have also shown that high MCT4 mRNA expression is associated with poor OS in bladder cancer patients (16). The expression of MCT4 protein was detected in several bladder cancer cell lines, and selective inhibition of MCT4 inhibited the viability of bladder cancer cells and reduced the tumor diameter of an orthotopic xenograft bladder cancer model (16). These studies highlighted the potential of MCT4 as a potential biomarker for bladder cancer.

In the present study, we first evaluated the expression of SLC16A3 mRNA in published single-cell RNA sequencing data of bladder cancer. Furthermore, the influence of SLC16A3 mRNA and MCT4 protein expression on the OS of bladder cancer patients was explored using the clinical data and MCT4 expression data of bladder cancer patients in our affiliations and The Cancer Genome Atlas (TCGA) database.

## Materials and Methods

### Acquisition of Publicly Available RNA Sequencing Data

The single-cell RNA sequencing data of eight bladder cancer samples and three normal bladder mucosa (NBM) samples were downloaded from the ENA database (https://www.ebi.ac.uk/ena/, accession ID: PRJNA662018) and Gene Expression Omnibus (https://www.ncbi.nlm.nih.gov/geo/, accession ID: GSE145140) (17, 18). The transcriptome data and survival data of 402 urothelial bladder cancer patients were obtained from TCGA database (https://tcga-data.nci.nih.gov/tcga/).

### Clustering and Cell-Type Annotation

Raw sequencing data were mapped to the human genome, and data cleaning and integration were performed using the Seurat package (19–21). Clustering analysis was performed using the “FindClusters” function of the Seurat package. Clusters were annotated to known cell types according to canonical cell-type-specific markers (22).

### Differential Analysis of Single-Cell Sequencing Data

The proportion of each epithelial cell cluster in all cells was calculated using the “prop.table” function of R software. The statistical significance of alterations in epithelial cell proportions was analyzed using one-way ANOVA and displayed with a histogram. The differential expression of SLC16A3 between epithelial cells of NMIBC and MIBC, as well as in NBM epithelial cells, was displayed using violin plots and t-SNE plots. The differentially expressed genes (DEGs) between SLC16A3-positive epithelial cells and SLC16A3-negative epithelial cells were screened using the “FindMarkers” function of the Seurat package with the default parameters. The DEGs were then submitted to Metascape software (https://metascape.org) for further Gene Ontology (GO) enrichment analyses.

### Patients and Samples of the Validation Cohort

From October 2010 to March 2015, 89 consecutive bladder cancer patients who had undergone radical cystectomy at Peking Union Medical College Hospital or Qingdao University Affiliated Hospital were enrolled. Formalin-fixed paraffin-embedded tumor samples were retrieved for further immunohistochemistry (IHC) analyses.

The inclusion criteria were as follows: 1) patients with urothelial cell carcinomas identified by pathologic examination and 2) patients who had undergone radical cystectomy for MIBC or high-risk NMIBC.

The exclusion criteria were as follows: 1) patients with other histological types of bladder tumors, such as squamous cell carcinoma or adenocarcinoma; 2) patients with insufficient tumor tissues for IHC analysis; and 3) patients with a history of cancers from other organs or systems.

Formalin-fixed paraffin-embedded tumor samples were retrieved for MCT4 IHC staining. The MCT4 expression of samples was scored according to the intensity of staining and ratio of positive cells (**Supplementary Method**).

### Statistical Analysis

Categorical variables were described with numbers and percentages in all cohorts. Continuous variables fitting a normal distribution were described with the mean ± standard deviation (SD). The association of MCT4 expression with categorical variables was assessed using chi-squared tests. Differences in continuous variables between two groups were analyzed using Student’s t-test. The statistical significance of alterations in epithelial cell proportions was analyzed using one-way ANOVA. The Kaplan–Meier (KM) method and log-rank test were used to analyze the influence of MCT4 expression on OS. Univariate Cox regression model was used to estimate the prognostic significance of each clinical characteristic and MCT4 expression. Variables that showed p < 0.1 in the univariate analysis were included in the multivariate Cox regression analysis. Variables that showed p < 0.05 in the multivariate analysis were independent prognostic factors for survival. The “nomogramEx” package of R software was used to draw a nomogram based on the results of the multivariate Cox regression. All p-values were two-sided, and statistical significance was defined as a p < 0.05. Unless otherwise noted, statistical analyses were performed using SPSS software (version 24.0).

## Results

### Clustering and Annotation of Single-Cell RNA Sequencing Data

**Figure 1:**
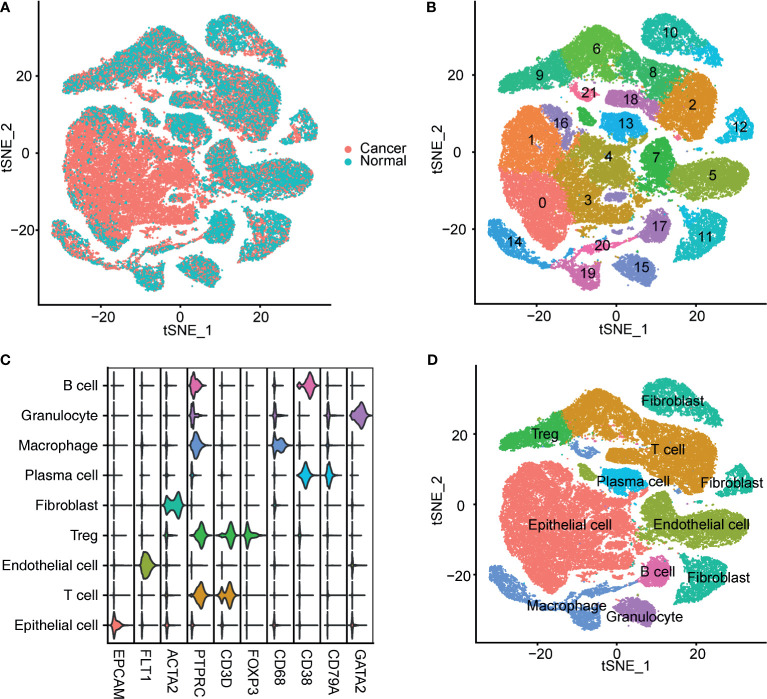
Identification of bladder cancer cells in single-cell sequencing data. **(A)** T-SNE plot of the integrated bladder cancer single-cell data. Each point represents a cell. Points were colored according to the pathological tissue type. **(B)** T-SNE plot of the cell clusters identified by the clustering algorithm. Points were colored according to the cluster identity. **(C)** Violin plot of the expression of classical markers of each cell type. **(D)** T-SNE plot of the annotated cell clusters. Each point represents a cell. Points were colored according to the cell type.

After preprocessing, 65,723 filtered cells were used for bioinformatics analysis, including 49,869 bladder cancer cells and 15,827 cells from the NBM (**Figure 1A**). Clustering analysis identified 22 cell clusters (**Figure 1B**), all of which were annotated according to the canonical cell-type markers (**Figure 1C**). Nine classical cell types were identified, including epithelial cells, endothelial cells, T cells, regulatory T cells (Tregs), fibroblasts, plasma cells, macrophages, granulocytes, and B cells (**Figure 1D**). Five cell clusters, including clusters 0, 1, 3, 4, and 16, were recognized as epithelial cells by high expression of epithelial cell adhesion molecule (EPCAM). Endothelial cells were marked by FLT1 expression. T cells were labeled according to their expression of CD3D. Tregs were identified by high CD3D and FOXP3 expression. Fibroblasts were labeled by ACTA2 expression. B cells and plasma cells were identified by CD38 and CD79A expression. Granulocytes were identified by GATA2, and macrophages were marked by CD68 expression (**Figure 1C**).

### Alteration of Epithelial Cells Between Bladder Cancer and the Normal Bladder Mucosa

**Figure 2:**
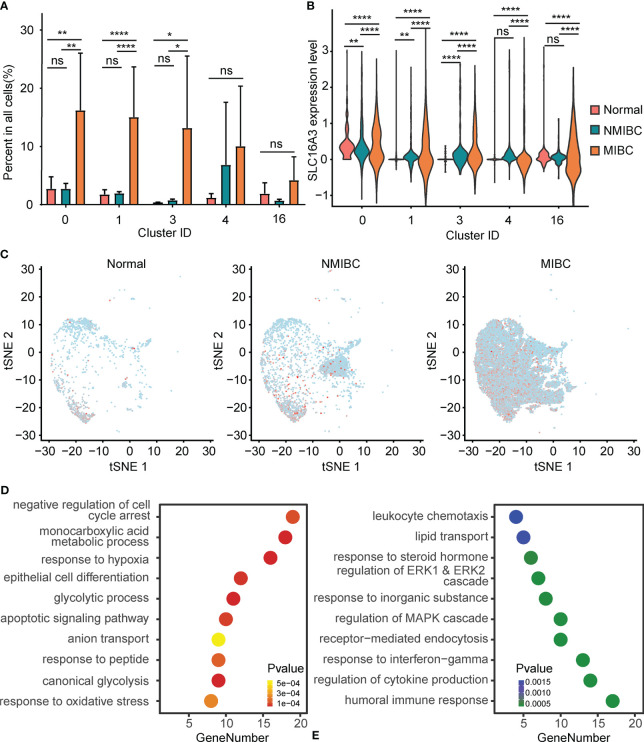
Alteration of PFKFB3 in bladder cancer cells. **(A)** Histogram of the percentage of epithelial cell adhesion molecule (EPCAM)-positive epithelial cell clusters in non-muscle invasive bladder cancer (NMIBC), MIBC, and the normal bladder mucosa. NS, not significant; *p < 0.05 **p < 0.01; ****p < 0.0001. **(B)** Violin plot of the expression of SLC16A3 mRNA in epithelial cell clusters of NMIBC, MIBC, and the normal bladder mucosa. NS, not significant; **p < 0.01; ****p < 0.0001. **(C)** T-SNE plot of the expression of SLC16A3 in epithelial cells in NMIBC, MIBC, and normal bladder mucosa. Red points represent cells with positive PFKFB3 expression, and gray points represent cells with no PFKFB3 expression. **(D)** Gene set enrichment analysis of the upregulated differentially expressed genes (DEGs) in SLC16A3-positive epithelial cells. The y-axis shows significantly enriched Gene Ontology (GO) terms, and the x-axis shows the number of DEGs enriched in a GO term. **(E)** Gene set enrichment analysis of the downregulated DEGs in SLC16A3-positive epithelial cells.

The percentages of epithelial cells in the NBM, NMIBC, and MIBC were 7.87%, 13.53%, and 73.20%, respectively. Clusters 0, 1, and 3 were significantly increased in MIBC, compared with those in the NMIBC and the NBM (**Figure 2A**). No statistically significant difference was found between the proportions of epithelial cell clusters of the NBM and NMIBC (**Figure 2A**).

Furthermore, compared with the NMIBC and the NBM, MIBC showed significantly elevated SLC16A3 mRNA in all epithelial cell clusters. Compared with the NBM, the NMIBC showed significantly elevated SLC16A3 mRNA in epithelial cell clusters 0, 1, and 3 (**Figure 2B**). Overall, a gradual increase could be found in the SLC16A3 mRNA expression of all epithelial cells in the NBM, NMIBC, and MIBC (**Figure 2C**).

Additionally, we screened DEGs between the SLC16A3-positive and SLC16A3-negative epithelial cells and found that 102 genes were upregulated and 66 genes were downregulated in SLC16A3-positive epithelial cells. The upregulated DEGs were mainly enriched in the glycolysis process pathway, response to hypoxia pathway, monocarboxylic acid metabolic process pathway, and epithelial cell differentiation pathway (**Figure 2D**). The downregulated DEGs were mainly enriched in regulation of humoral immune response pathway and MAPK cascade pathway (**Figure 2E**).

### Prognostic Significance of SLC16A3 mRNA Expression in Bladder Cancer

**Figure 3:**
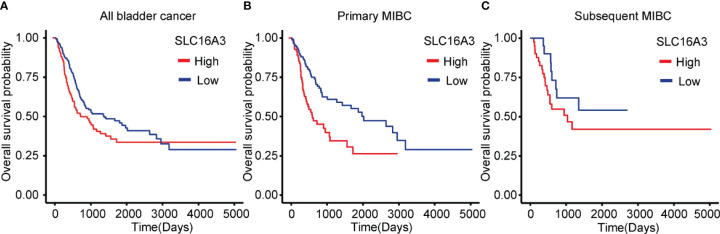
Influence of SLC16A3 expression on the survival of bladder cancer patients. **(A)** Bladder cancer patients with high SLC16A3 mRNA expression had worse overall survival rates than those with low SLC16A3 mRNA expression (p = 0.016). **(B)** Primary muscle invasive bladder cancer (MIBC) patients with high SLC16A3 mRNA expression showed worse overall survival rates than those with low SLC16A3 mRNA expression (p < 0.001). **(C)** Subsequent MIBC patients with high SLC16A3 mRNA expression showed no significant difference from patients with low SLC16A3 mRNA expression (p = 0.204).

To explore the influence of SLC16A3 mRNA expression on the survival of bladder cancer patients, we downloaded the survival and transcriptome sequencing data of 402 patients in TCGA database, and the KM survival analysis showed that the patients with high SLC16A3 mRNA expression showed a significantly poorer OS than those with low SLC16A3 mRNA expression (p = 0.012; **Figure 3A**). Furthermore, subgroup analysis of 234 primary MIBC patients showed that primary MIBC with high SLC16A3 mRNA expression revealed a significantly worse OS (p < 0.001; **Figure 3B**). Subgroup analysis of 168 subsequent MIBC with an NMIBC history showed that tumors with different SLC16A3 mRNA expression levels did not show a significant difference (p = 0.204, **Figure 3C**).

### Clinical Characteristics and MCT4 Expression of the Validation Cohort

We further enrolled a validation cohort including 89 consecutive bladder cancer patients. The average age of these patients was 69.1 years, and 50 of them had a smoking history. Seventy-two of them had MIBC, and the remaining 17 patients had high-risk NMIBC. Ten patients had previously received intravesical pirarubicin or Bacillus Calmette–Guérin vaccine therapy. Fifty-nine of them had one tumor focus. The other 30 patients had multiple tumors. The average number of tumors was 1.9, and the average maximum tumor diameter was 4.3 cm. Seventy-six tumors showed low pathological differentiation, while 13 tumors showed high differentiation. Pathological vessel or nerve invasion was found in tumors from 11 and eight patients, respectively. The median follow-up time was 33.2 months (**Table 1**). No patient died from preexisting chronic disorders during the follow-up.

**Table 1:** Baseline information.

Gender	Male	76
	Female	13
Age (year)		69.1 ± 10.6
Differentiation	Low	76
	High	13
Muscle infiltration	No	17
	Yes	72
Vessel invasion	No	78
	Yes	11
Nerve invasion	No	81
	Yes	8
History of smoking	No	39
	Yes	50
Intravesical therapy	No	79
	Yes	10
Maximum tumor diameter (cm)		4.3 ± 1.9
Number of tumors		1.9 ± 1.5
Follow-up time (month)		33.2 ± 19.4

**Figure 4:**
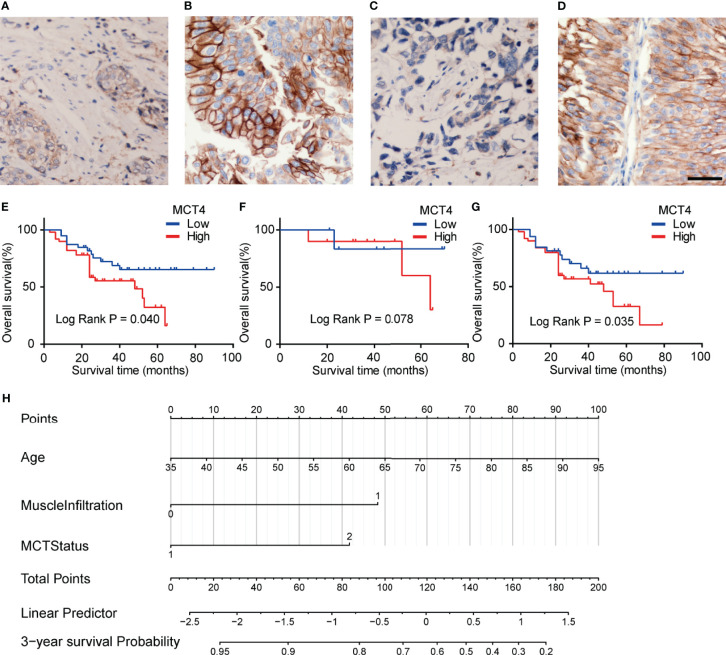
Expression of MCT4 protein in bladder cancer. Low MCT4 **(A)** and high MCT4 **(B)** expression in non-muscle invasive bladder cancer (NMIBC). Low MCT4 **(C)** and high MCT4 **(D)** expression in MIBC. Magnification: ×400. Scale bar: 10 μm. **(E)** High MCT4 protein expressions were associated with poor overall survival (OS) in all bladder cancer patients who received radical cystectomy (log-rank test, p = 0.040). **(F)** No significant difference was found between the OS of NMIBC patients with high MCT4 protein expression and NMIBC patients with low MCT4 expression (log-rank test, p = 0.078). **(G)** MIBC patients with high MCT4 protein expressions have worse OS rates than those with low MCT4 expression (log-rank test, p = 0.035). **(H)** Nomogram to predict the postcystectomy survival of bladder cancer patients. Patients were scored according to age (0–100 scores), existence of MIBC (48 for MIBC and 0 for NMIBC), and MCT4 expression (0 for low MCT4 expression and 42 for high MCT4 expression). The scores were then summed to obtain the total points; and the number in the last line, which is vertically beneath the accumulated total points, is the predicted 3-year survival for this patient.

IHC staining was used to examine the expression of MCT4 protein in bladder cancer samples. Positive staining was mainly found in the membrane of the cancer cells, while the nuclei were not stained (**Figures 4A–D**). Fifty tumor tissues showed high MCT4 expression, and the remaining 39 tumors showed low MCT4 expression. Patients with different gender, pathological characteristics, smoking history, intravesical therapy, tumor diameter, or tumor numbers showed no significant difference in MCT4 expression (**Table 2**).

**Table 2:** MCT4 expressions and clinical characteristics.

MCT4 expression		Low	High	p-Value^*^
Gender	Male	33	43	0.854
	Female	6	7	
Differentiation	Low	35	41	0.305
	High	4	9	
T stage	T1	7	10	0.978
	T2a	13	14	
	T2b	17	23	
	T3	1	2	
	T4	1	1	
Muscle infiltration	No	7	10	0.807
	Yes	32	40	
Vessel invasion	No	34	44	1
	Yes	5	6	
Nerve invasion	No	37	44	0.458
	Yes	2	6	
History of smoking	No	17	22	0.969
	Yes	22	28	
Intravesical therapy	No	35	44	1
	Yes	4	6	
Maximum tumor diameter (cm)		4.37 ± 2.15	4.15 ± 1.64	0.589
Number of tumors		1.85 ± 1.41	1.98 ± 1.61	0.682

^*^The difference in categorical characteristics between bladder cancer with low and high MCT4 expression was analyzed using chi-squared test. The differences in numeric characteristics that were normal distribution were analyzed using Student’s t-test.

### Prognostic Significance of MCT4 Expression

The KM analysis showed that high MCT4 protein expression was associated with poor OS after cystectomy in bladder cancer patients (p = 0.04; **Figure 4E**). In the subgroup analysis of NMIBC patients, no significant difference was found in OS between tumors with high and low MCT4 expression (p = 0.078; **Figure 4F**), while in the MIBC patients, tumors with high MCT4 expression showed significantly worse OS than those with low MCT4 expression (p = 0.035; **Figure 4G**).

Furthermore, in the univariate Cox regression analysis, high MCT4 expression (HR: 2.024; 95% CI: 1.004–4.082; p = 0.049) and nerve infiltration (HR: 2.801; 95% CI: 1.153–6.804; p = 0.023) showed a significant influence on the OS of bladder cancer patients (**Table 3**). Characteristics with p-values <0.1, including age, MCT4 expression, nerve invasion, and muscle infiltration, were further analyzed in the multivariate Cox regression model. High MCT4 expression (HR: 2.462; 95% CI: 1.202–5.042; p = 0.014) and age (HR: 1.037; 95% CI: 1.002–1.073; p = 0.037) were independent predictors for the postcystectomy OS of bladder cancer patients (**Table 3**). To facilitate the use of this model, we built a nomogram for postcystectomy survival prediction using the results of Cox regression analysis (**Figure 4H**).

**Table 3:** Univariate and multivariate Cox regression.

	Univariate analysis			Multivariate analysis	
	HR (95% CI)		p-Value	HR (95% CI)	p-Value
Age	1.031 (0.997–1.067)		0.074	1.037 (1.002, 1.073)	0.037
Gender	2.344 (0.718–7.655)		0.158		
Tumor size	1.065 (0.916–1.238)		0.412		
Tumor number	0.858 (0.669–1.100)		0.228		
Muscle infiltration	0.399 (0.14–1.135)		0.085	0.348 (0.119, 1.011)	0.052
Vessel invasion	1.547 (0.642–3.729)		0.331		
Nerve invasion	2.801 (1.153–6.804)		0.023*	2.149 (0.869, 5.315)	0.098
History of Smoking	0.91 (0.473–1.753)		0.779		
Intravesical therapy	1.438 (0.558–3.706)		0.451		
MCT4 expression	2.024 (1.004–4.082)		0.049*	2.462 (1.202–5.042)	0.014

## Discussion

Bladder cancer is the 10th most common cancer and most common urothelial malignancy worldwide, with approximately 573,000 new cases and 213,000 deaths per year (1). However, 26.9%–37.5% of patients who receive radical treatment show tumor recurrence or metastasis (3–5). Recent advances in prognostic biomarkers provided new methods for more individualized surveillance and treatment of bladder cancer (23, 24). Comprehensive use of cystoscopy, liquid biopsy, and pathology examination could profoundly facilitate the diagnosis and surveillance of bladder cancer and could improve the survival of bladder cancer patients (23, 25).

Despite substantial advances in the field of biomarker detection, new cost-effective markers are still needed to improve diagnostic accuracy and reduce further testing (25). The reprogramming of energy metabolism is a canonical hallmark of cancer cells. Cancer cells rely on glycolysis to generate ATPs even in the presence of oxygen. This phenomenon is termed aerobic glycolysis or the Warburg effect (26). In tumor cells, aerobic glycolysis can rapidly generate ATP and lactate. The lactate produced by aerobic glycolysis plays an important role in the immune evasion and tumor angiogenesis (9, 27). Enhanced lactate shuttling between the cytoplasm and microenvironment is needed to induce immune evasion and tumor angiogenesis and to maintain the stable intracellular pH of tumor cells (28). MCT4 is the major type of transmembrane lactate transporter because of its high preference for lactate. MCT4 inhibition results in intracellular accumulation of lactate, reduction in cell growth and tumor angiogenesis, and induction of reactive oxygen species generation and apoptosis (9, 16). High MCT4 expression is also a signal for poor response to platinum-based chemotherapy (29, 30). In this study, we evaluated the possible use of MCT4 as a potential prognostic biomarker for bladder cancer patients.

The bladder cancer microenvironment is rich in various mesenchymal cells (31). The complex microenvironment is an obstacle for the study of glycolytic tumor biomarkers. In polymerase chain reaction (PCR), bulk RNA sequencing, and Western blotting assays, the upregulation of MCT4 in cancer cells may be covered by other cells with high MCT4 expression, such as immune cells and vascular endothelial cells. Single-cell sequencing is an emerging technique in cancer research that allows examination of the alteration of cancer cells alone. In this study, using single-cell RNA sequencing data, we identified five clusters of epithelial cells, three of which were enriched in MIBCs. Although the proportions of these five clusters were similar between the NMIBC and the NBM, SLC16A3 mRNA was upregulated in three clusters of epithelial cells in the NMIBC compared with the NBM. Additionally, the expression of SLC16A3 was markedly upregulated in all five epithelial cell clusters in MIBC compared with both the NMIBC and the NBM. These results suggest that the upregulation of MCT4 may occur earlier than the massive proliferation of malignant epithelial cells in the development of bladder cancer. More importantly, DEG screening and GO enrichment analysis showed that glycolytic processes and monocarboxylic acid metabolic processes were upregulated in SLC16A3-positive cells, which proves that the expression of SLC16A3 mRNA is closely related to aerobic glycolysis pathways. Therefore, SLC16A3 expression could well reflect the activation status of tumor aerobic glycolysis.

In this study, survival analysis of the transcriptome and the clinical data of bladder cancer patients in TCGA database showed that high SLC16A3 mRNA expression was associated with poor OS in patients with primary MIBC. SLC16A3 mRNA expression showed only a statistically insignificant trend of different OS in subsequent MIBC with NMIBC history. The time needed to progress to MIBC from the NMIBC is highly variable because of the heterogeneity in NMIBC malignancy, likely a reason for this insignificant result.

Furthermore, the KM analysis of the clinical and IHC data of the validation cohort showed that high MCT4 protein expression was significantly associated with poor OS in bladder cancer patients. Subgroup analysis showed that MIBC patients with high MCT4 expression had significantly worse OS than those with low MCT4 expression. In the NMIBC patients, the KM analysis showed insignificant results (p = 0.072), which may be caused by the small NMIBC patient number. However, a trend of poor OS in patients with high MCT4 expression was still found in the KM plot. Choi et al. (32) also reported that MCT4 protein expression negatively correlates with recurrence-free survival in bladder cancer patients in a cohort including all stages of bladder cancer. However, in their study, the therapy for bladder cancer was not specified. Since treatment and tumor malignancy potential can vary considerably based on the TNM stage of bladder cancer, the lack of treatment information may lead to intervention-derived bias of the results. Compared with the cohort reported by Choi et al., we enrolled only patients who had undergone radical cystectomy. Our cohort is more homogeneous in clinical treatment; thus, our results can better support using MCT4 as a prognostic biomarker in MIBC.

Finally, the multivariate Cox regression revealed that high MCT4 expression and age are independent prognostic factors for poor OS in bladder cancer patients undergoing cystectomy. Overall, we found that MCT4 IHC staining can help identify bladder cancer patients with high postcystectomy mortality. We built a nomogram according to the results of Cox analysis. Each patient was scored according to age, the existence of MIBC, and the MCT4 expression status. The scores were then summed to obtain the total points, which were used to predict the 3-year OS for each patient (**Figure 4H**). Based on the nomogram, the influence of MCT4 expression on the survival of bladder cancer patients can be considered along with other regular prognostic factors, such as age and the depth of invasion. Patients with a poor OS prediction should receive more frequent surveillance after cystectomy, and adjuvant chemotherapy and radiotherapy could be considered based on the prediction of the nomogram and clinical status of the bladder cancer patients. Additionally, targeting MCT4 inhibits the viability of bladder cancer cells and reduces the tumor diameter in a xenograft bladder cancer tumor model (9, 16). Thus, bladder cancer patients with high MCT4 expression may also benefit from MCT4-targeted therapy. Additionally, the use of MCT4 targeted therapy may be combined with functional MRI or PET-CT imaging to screen *in vivo* drug dynamics (33–37). Furthermore, the combination of MCT4 IHC and previously identified noninvasive peripheral blood biomarkers, such as survivin, circulating tumor cells, and systemic combining inflammatory score, may also help to further improve the clinical management of bladder cancer (38–41). However, further preclinical and clinical studies are needed to further validate the effect of MCT4-targeted therapy in bladder cancer.

Our studies have some limitations. First, all samples were collected during radical cystectomy; thus, we lacked data on MCT4 protein expression in early-stage bladder cancer and metastatic bladder cancer, which are not eligible for radical cystectomy. This finding may limit the use of nomograms in the NMIBC and metastatic bladder cancer patients. Second, chronic disorders such as diabetes mellitus and obesity may also significantly influence the survival of bladder cancer patients (42–44). In this study, the influence of preexisting chronic disorders on the survival of bladder cancer patients could not be ruled out. Further studies with more detailed background information and larger sample sizes are needed to better use MCT4 in the prognosis of bladder cancer.

In conclusion, our study revealed that SLC16A3 mRNA is upregulated in bladder cancer cells. Bladder cancer with high SLC16A3 mRNA expression has a poor OS. High MCT4 protein expression is an independent prognostic factor for bladder cancer patients who had undergone radical cystectomy, and a nomogram based on the Cox regression analysis of our data was built to facilitate the use of MCT4 expression in the prognosis of bladder cancer.

## Data Availability Statement

The raw data supporting the conclusions of this article will be made available by the authors, without undue reservation.

## Ethics Statement

The studies involving human participants were reviewed and approved by the ethics committee of Peking Union College Hospital and the ethics committee of Affiliated Hospital of Qingdao University. The patients/participants provided their written informed consent to participate in this study.

## Author Contributions

YZ, BZ, and Y-SZ analyzed the data and drafted the manuscript. YX, and W-HY performed the IHC staining and scoring. YZ, BZ, Z-HW, W-DW, G-YZ, and Y-SZ collected the tumor samples and performed the follow-up of patients. All authors contributed to the article and approved the submitted version.

## Funding

This study was funded by the grants from the National Natural Science Foundation of China (grant no. 81602231 and 81670611) and Major Research Program of Shandong Province (grant no. 2017GSF221016).

## Conflict of Interest

The authors declare that the research was conducted in the absence of any commercial or financial relationships that could be construed as a potential conflict of interest.

## Publisher’s Note

All claims expressed in this article are solely those of the authors and do not necessarily represent those of their affiliated organizations, or those of the publisher, the editors and the reviewers. Any product that may be evaluated in this article, or claim that may be made by its manufacturer, is not guaranteed or endorsed by the publisher.
