# Supplementary material for: Integrative Analysis Identified MCT4 as an Independent Prognostic Factor for Bladder Cancer
Source: Front Oncol. 2021 Aug 26;11:704857. doi: 10.3389/fonc.2021.704857 (PMC8426349; doi:10.3389/fonc.2021.704857)
Supplement: Supplementary file 1 [file DataSheet_1.docx]

**Supplementary methods:**

**Pre-processing of raw single-cell sequencing data.**

Raw sequencing data were mapped to the human genome (GRCh38) through Cell Ranger toolkit (version 2.1.1) to generate gene expression matrix. The UMI count data from all samples were read into the Seurat package (version 3.2.2) in R software (version 4.0.3) for further processing. Cells which expressed 200 to 2500 genes, and had less than 10 percent mitochondrial gene content were used for further analysis. Each UMI count matrix was then scaled by library size and log-transformed. The top 3000 highly variable genes were identified through the function “FindVariableGenes” in Seurat package. Then all the datasets were integrated using the “FindIntegrationAnchors” and “IntegrateData” functions in Seurat package.

**IHC staining**

Formalin-fixed, paraffin-embedded tissues were cut into 4 μm sections, deparaffinized, and rehydrated using ethanol. Sections were heated to 121°C in 1 mmol/L EDTA, and then cooled to 90°C, and incubated for 5 minutes, and treated with peroxidase blocking reagent (Dako, Denmark) to retrieve antigens. Then the sections were incubated in 1:100 diluted polyclonal rabbit anti-MCT4 primary antibody (Sigma-Aldrich, Cat HPA012451, USA) for 10 minutes at room temperature, followed by rabbit anti-mouse HRP-labeled antibody (Solarbio, China) for 2 hours, and colored using a DAB Horseradish Peroxidase Color Development Kit (Beyotime, China) for 30 min. Sections were then lightly counterstained with hematoxylin.

**IHC scoring**

Stained sections were evaluated by two independent pathologists who did not acknowledge the clinical information of the patients. Each section was scored according to the intensity of staining and the ratio of positive cells: Sections with no staining were scored by 0 point; sections with light yellow staining were scored with 1 point, sections with light brown staining were scored with 2 points, and sections with dark brown staining were scored with 3 points. Sections with less than 5% positive cells were scored with 0 point, sections with 6-25% positive cells were scored with 1 point, sections with less than 26-50% positive cells were scored with 2 points, sections with 51-75% positive cells were scored with 3 points, and sections with more than 75% positive cells were scored with 4 points. The MCT4 scores were achieved by multiplying the staining intensity scores and the positive cell ratio scores. Sections with more than 6 scores were defined as high MCT4 expression, otherwise low MCT4 expression.
